# Hummingbird and Hawkmoth Wing Shape: Analyzing Functional Convergence in Analogous Structures

**DOI:** 10.1093/icb/icag088

**Published:** 2026-06-16

**Authors:** Benton Walters, Yuming Liu, Iman Fadel, Humberto G Ferrón, Emily J Rayfield, Philip C J Donoghue

**Affiliations:** Bristol Palaeobiology Group, School of Earth Sciences, University of Bristol, Bristol BS8 1TQ, UK; Bristol Palaeobiology Group, School of Earth Sciences, University of Bristol, Bristol BS8 1TQ, UK; Bristol Palaeobiology Group, School of Earth Sciences, University of Bristol, Bristol BS8 1TQ, UK; Cavanilles Institute Biodiversity and Evolut. Biol. (ICBBE), Universitat De València, Valencia 46980, Spain; Bristol Palaeobiology Group, School of Earth Sciences, University of Bristol, Bristol BS8 1TQ, UK; Bristol Palaeobiology Group, School of Earth Sciences, University of Bristol, Bristol BS8 1TQ, UK

## Abstract

Similar wing planform shapes have independently evolved in both hummingbirds and hawkmoths, indicating convergence. Functional analysis of bird wings has shown that the rigours of flight are a constraining influence on shape, resulting in evolution toward better functioning forms. Shape similarity in these analogous structures may therefore represent convergence upon a more functionally adapted morphology. Using a theoretical morphospace, we test the performance of hummingbird and hummingbird-mimicking moth wings and compare with birds and a dataset of other insect groups. When compared with other birds, hummingbirds demonstrate a strong functional constraint, though this effect is absent when compared with insects that more closely match their flight mode. The phylogenetic signal of hummingbird wing shape is minimal, though this result is likely clouded by variation introduced by sexual dimorphism. Evidence of functional constraint in the sampled planforms is small and varies according to taxa, with *Macroglossum* moths exhibiting the least constraint. This suggests that morphological convergence between hawkmoths and hummingbirds may result from selective pressures external to the functional constraints of flight.

## Introduction

Of all vertebrates, hummingbirds are among the most highly specialized fliers. The constraints of flight have extensively moulded the hummingbird Bauplan, affecting overall body size and metabolic rate, and leading to a drastic reduction in lower limb size and the adoption of a bidirectional wing beat cycle, similar to that used by insects (Pearson 1950; Suarez 1992; Tobalske 2010; Warrick et al. 2012b; Ingersol and Lentink 2018). Both *Hemaris* and *Macroglossum* hawkmoths have adopted similar strategies to hummingbirds, and their wing shape is an example of convergence in analogous structures (Halloway et al. 2018; Amorim 2020). Given the intensity of flight, adaptationist reasoning posits this highly specialized morphology results from functional constraint, with resulting similarity in shape arising from convergence upon an optimal form (Gould and Lewontin 1979). Shared development of a hummingbird wing planform shape therefore represents a potential example of functionally driven convergence in shape between animals from different branches of the tree of life.

Across birds there is evidence for function-based constraint, particularly within hummingbirds, which show a high degree of optimization for a combination of functional metrics associated with lift generation and aerial agility (Walters et al. 2026). Due to the substantial changes to the hummingbird body plan, and their insect-like kinematics, comparing hummingbirds with exclusively avian taxa paints an incomplete picture of the link between wing shape and performance in these animals. Analysis of hummingbird wing shape requires functional comparison with both vertebrate and insect wings. Other factors such as sexual dimorphism and phylogenetic relationships could provide alternative non-functional constraints on shape. In several hummingbird tribes, such as *Mellisuginae*, males possess wings modified to produce sound for display and are demonstrated to fly differently than females in experimental conditions (Chai et al. 1999; Stiles et al. 2005; Clark and Dudley 2009; Segre et al. 2016; Wilcox and Clark 2022). To isolate the role that flight performance plays in constraining shape, and how this may drive convergence with moths, a method is needed to directly link morphological change with relative performance.

Theoretical morphospace analyzes a global environment of shape, providing a valuable method for investigating the relationship between form and function (McGhee 2006). Shapes are generated to produce an even grid of variance in form that exceeds that realized by evolution, in effect producing a shape space that encompasses all possible variation, irrespective of whether this shape has evolved in nature. Calculating performance for a single metric across all theoretical shapes produces a performance surface, where the highest value shape can be said to represent the global optimum of form for that metric (Deakin et al. 2022; Walters et al. 2026). Pareto ranking algorithms combine multiple discrete functional surfaces to examine trade-offs that more closely resemble the pressures affecting complex animal behaviors like flight (Deakin et al. 2022; Walters et al. 2026). Once this space is established and optima are identified, empirical taxa can be projected into the landscape and their relative functional performance deduced through proximity to the peak. Any taxa that overlap with a peak can be said to embody high functional constraint (Deakin et al. 2022). This method has already been used to study the form of wings in both birds and insects (Liu et al. 2024; Fadel et al. 2025; Walters et al. 2026) and is especially valuable here. Since the analysis is performed on theoretical rather than empirical shapes, form can be examined across non-homologous structures, thus sidestepping a key issue of comparing these two groups.

Prior analysis of wing form and function demonstrates the significant role that planform shape plays across a variety of flight modes with the development of specialized wings for specific flight styles (Saville 1957; Rayner 1988; Taylor and Thomas 2014; Walters et al. 2026). This builds on a substantial body of research into the mechanics of avian flight, linking the function of the wing with aerodynamic principles (Pennycuick 1975, 1989; Warham 1977; Norberg and Rayner 1987; Norberg 1987, 1990, 2002) and discussion of how specific aspects, such as wingtip shape affect flight performance (Lockwood et al. 1998; Swaddle and Lockwood 2003). Hummingbird flight, in particular, has been extensively researched, with special focus on understanding the kinematics of bidirectional lift generation from their insect-like wingbeat through modeling and *in vivo* analysis (Warrick et al. 2005, 2009, 2012a; Tobalske et al. 2007; Ingersol and Lentink 2018; Haider et al. 2021).

Here, we use the combination of lift generation and agility, metrics pertaining to forward flight that have been previously suggested as an optimizing force on hummingbird wings by Walters et al. (2026). In hummingbird wing shape space, we find an antagonistic trade-off between these two metrics and propose that, if hummingbird wing planforms are highly constrained by function, as previously found, their planforms should overlap with the peak for this trade-off. Strong functional constraint should exist both when viewed independently, and in a broader shape space including multiple insect groups. Occupation of shared space between hummingbirds and *Hemaris* and *Macroglossum* moths would therefore indicate independent evolutionary convergence in shape due to functional constraint, as proposed by adaptationist thinking.

## Methods

### Data collection

Hummingbird wing planform outlines were collected from images, primarily of spread specimens in the collection of the Burke Museum of Natural History and Culture (UWBM), with supplemental collection from published resources. All wings were photographed from directly above to accurately capture planform shape and, where possible, right wings were selected to improve uniformity across the dataset. Where known, sex was recorded to permit analysis of potential dimorphism. Wings for both *Hemaris* and *Macroglossum* moths were procured from photographs produced by Didier Descouens from private collections, accessed under creative commons license CC BY-SA 4.0. Both forewings and hindwings were collected as a single unit, as this best simulates their use in flight and kinematic modeling (Warfvinge et al. 2021; Lionetti et al. 2022; Xue et al. 2024), providing a more comparable wing planform to hummingbirds. A complete taxa list and attribution is available in the Supplementary Material (Data 1) as well as the outlines used in the analysis (Data 3).

**Fig. 1:**
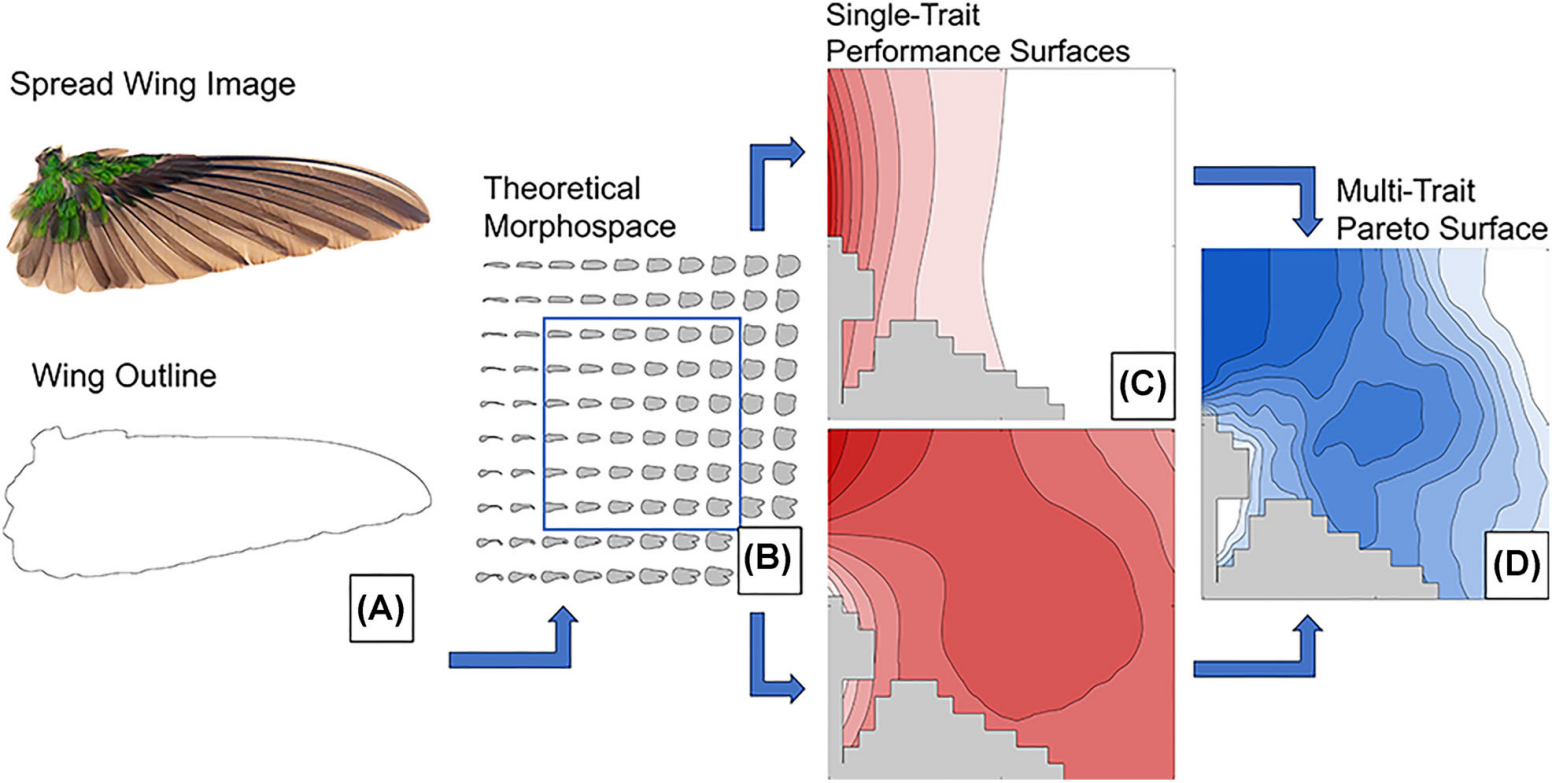
Theoretical morphospace construction and functional testing workflow. (A) characterization of empirical planform shape by EFA to produce a single pixel outline. (B) generation of theoretical planform shapes, boxed shapes indicate the extent of empirically realized morphology. (C) generation of single trait performance surfaces through functional testing of theoretical planform shapes. (D) combination of multiple single trait surfaces through Pareto ranking to produce an optimality surface.

Additional datasets were sourced for Hymenoptera, Neuroptera, and Lepidoptera wing plan forms to cover a diversity of insect groups with varying flight modes (Liu et al. 2024; Fadel et al. 2025). Planform outlines were extracted from wing images using the outline function in FIJI version 1.54 (Schindelin et al. 2012), to produce single pixel outlines suitable for Elliptical Fourier Analysis (EFA) (Fig. 1A). Subsequent analysis was conducted in MATLAB version R2022b (Mathworks 2022). Fourier shape analysis was performed using the method outlined by Deakin et al. (2022), utilizing 1200 outline landmarks and 20 shape harmonics, the maximum number possible given the resolution of the original images. All wing planforms and generated theoretical shapes were standardized for size using the first elliptic, thus isolating the effect of shape in the dataset.

### Phylogenetic analysis and sexual dimorphism

A time-scaled tree of all sampled hummingbird taxa was constructed from a subset of the birdtree.org supertree, using the Hackett backbone (Hackett et al. 2008). Least squares strict consensus was achieved using the consensus edges tool in the R package phytools (Revell 2011). Phylogenetic signal in the dataset was tested using the physignal command in the R package geomorph (Adams and Otárola-Castillo 2013 ). This produces a multivariate K statistic (kmult) of the relationship between phylogeny and shape. All files used to produce the consensus tree and calculation of phylogenetic signal are available in the Supplementary Material (Data 4 and Code 1). To investigate the effects of sexual dimorphism, hummingbird planforms were plotted in a principal components space and color coded according to sex. This was essential as both male and female specimens were included in the dataset. If sexual dimorphism drives variation within the dataset, it would be expected that male hummingbirds belonging to *Mellisugini* would plot eccentric to the majority of the sampled taxa.

### Theoretical morphospace

For all theoretical morphospaces, hummingbirds and both *Hemaris* and *Macroglossum* moths were combined into a single dataset to analyze their relative performance. Two parallel analyses were performed, with and without the additional three insect groups. Creation of theoretical shapes followed the method outlined by Deakin et al. (2022) and adapted for bird wing planforms by Walters et al. (2026). Variation in empirical planform shapes was first characterized by principal components analysis (PCA) and then the shape parameters of the harmonic formulae sequentially altered to produce an even grid of theoretical shapes (Fig. 1B). Theoretical shapes could be generated that exceed the bound of empirically realized variation by 20%, producing an even grid of 520 (20 × 26) shapes for hummingbirds and moths on their own and 544 (17 × 32) for the other insect groups. Expansion of theoretical space beyond that occupied by nature allowed for identification of potential optimal morphologies not realized in living animals. Where theoretical shapes self-intersected, this region was considered impossible space. These shapes were removed from analysis and are represented by grey boxes in performance surface plots (Deakin et al. 2022; Walters et al. 2026).

### Performance proxy

This study examines relative flight performance for highly mobile forward flight. To calculate flight performance, two metric proxies were used: second moment of area and pitch agility. These metrics were chosen from previous bird-specific research, which suggested that they were a constraining factor on hummingbird wings (Walters et al. 2026). This provides a useful point to compare with hummingbird-mimicking moths and other insects. While this metric combination does not describe hovering flight, it does pertain to highly agile, energy-intensive flight, and permits continuity between analyses.

The second moment of area of each theoretical shape corresponds to the distribution of the aerodynamic surface along its length (Ellington 1984; Hally 1987; Liu et al. 2024; Walters et al. 2026). Second moment of area relates to both the lift generation potential of planforms as well as manoeuvrability about the *x*-axis, traits that are advantageous for energy-intensive, highly mobile flight. Performance scores for this metric were calculated following the method outlined by Liu et al. (2024). Pitch agility relates to the ability to generate angular acceleration around the *y*-axis of span (Harvey et al. 2022). Higher agility allows the bird to more quickly pitch up and down in flight and was calculated using the formula produced by Harvey at al. (2022), adapted for use with theoretical shapes by Walters et al. (2026).

**Fig. 2:**
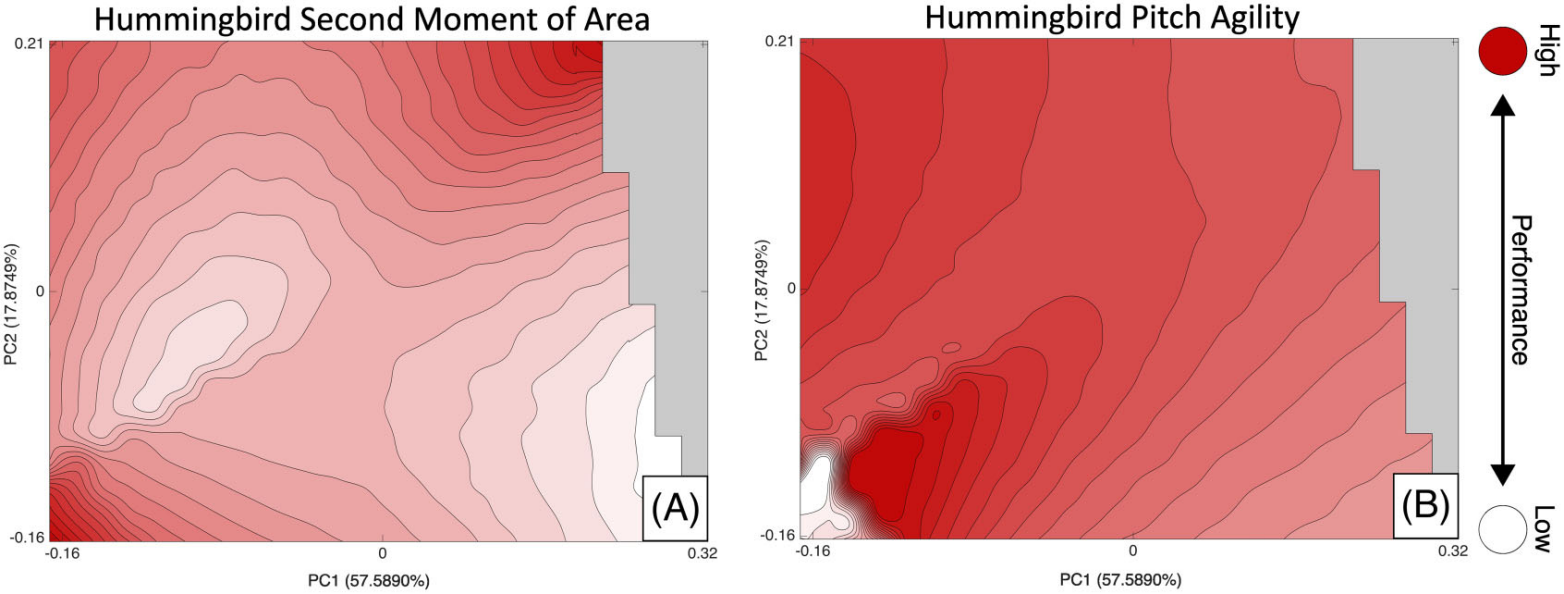
(A) single trait performance surface for second moment of area, calculated for theoretical hummingbird and hawkmoth shapes. (B) single trait performance surface for pitch agility. Areas of low performance in second moment of area correspond with high performance in pitch agility, indicating an antagonistic trade-off.

Each theoretical shape was tested for performance in the two proxies, producing a grid of relative values, with the highest-scoring theoretical shape interpreted to be the most optimal for that metric (Fig. 1C). An antagonistic trade-off was identified in some areas of theoretical space, indicating that relative performance for one metric conversely affects performance for the second (Fig. 2). To analyze constraint for this trade-off, single-trait performance surfaces were combined to produce a singular ‘optimality surface’, utilizing a Goldberg Pareto ranking algorithm developed by Deakin et al. (2022; Goldberg 1989). This method has been demonstrated as an effective means of analyzing complex performance interactions in morphospaces (Stayton 2019, Dickson and Pierce 2019; Dickson et al. 2021; Liu et al. 2024; Rawson et al. 2024; Berks et al. 2025; Fadel et al. 2025; Griffin et al. 2025; Walters et al. 2026). The resulting surface ranks theoretical shapes from 0 to 1, based on the trade-off between the two tested metrics, with 1 representing the most optimal (Fig. 1D). All executable files used in the analysis are available in the Supplementary Material (Data 2 and Code 1).

## Results

### Non-function constraints

**Fig. 3:**
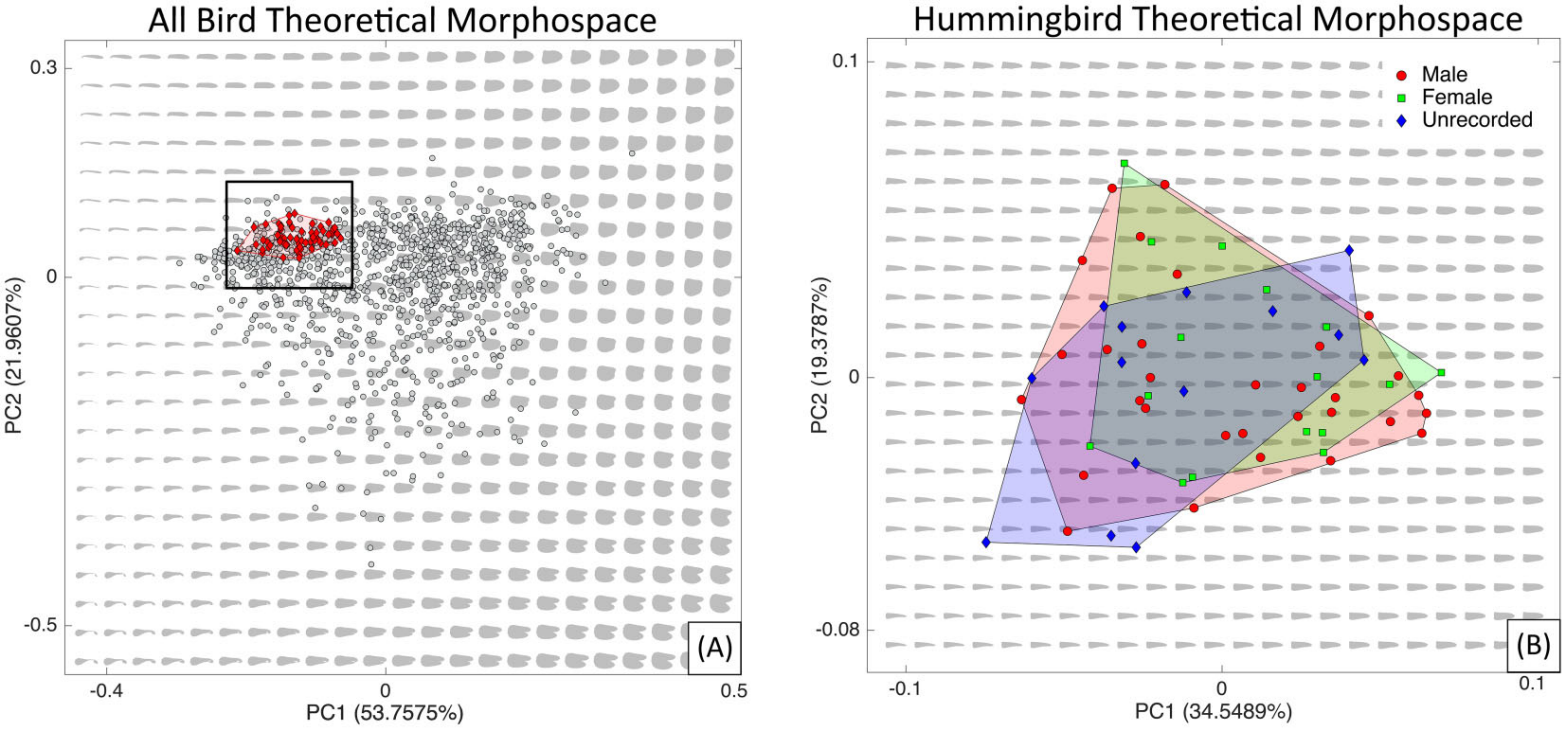
(A) Theoretical morphospace of all birds adapted from Walters et al. (2026) with sampled hummingbird taxa (diamonds) highlighted in red. (B) principal components analysis of sampled hummingbird wing planforms, colored according to sex.

Compared with all birds (Fig. 3A), hummingbirds occupy a constrained area of shape space which overlaps a densely occupied region. The first two principal components of hummingbird planform variation (Fig. 3B) encompass approximately 54% of variation in shape. Space occupation between males and females overlaps almost entirely, with the convex hull for female wing planforms encompassing a greater extent of morphospace. The most eccentric planform belongs to the Black-throated Mango (*Anthracothorax nigricollis*) specimen of unrecorded sex. There is no recognisable difference in the distribution of male and female *Mellisugini* hummingbirds. Phylogenetic signal within the hummingbird dataset is low and not found to be significant with a Kmult of 0.2186 (*P* = 0.067).

### Theoretical shape

**Fig. 4:**
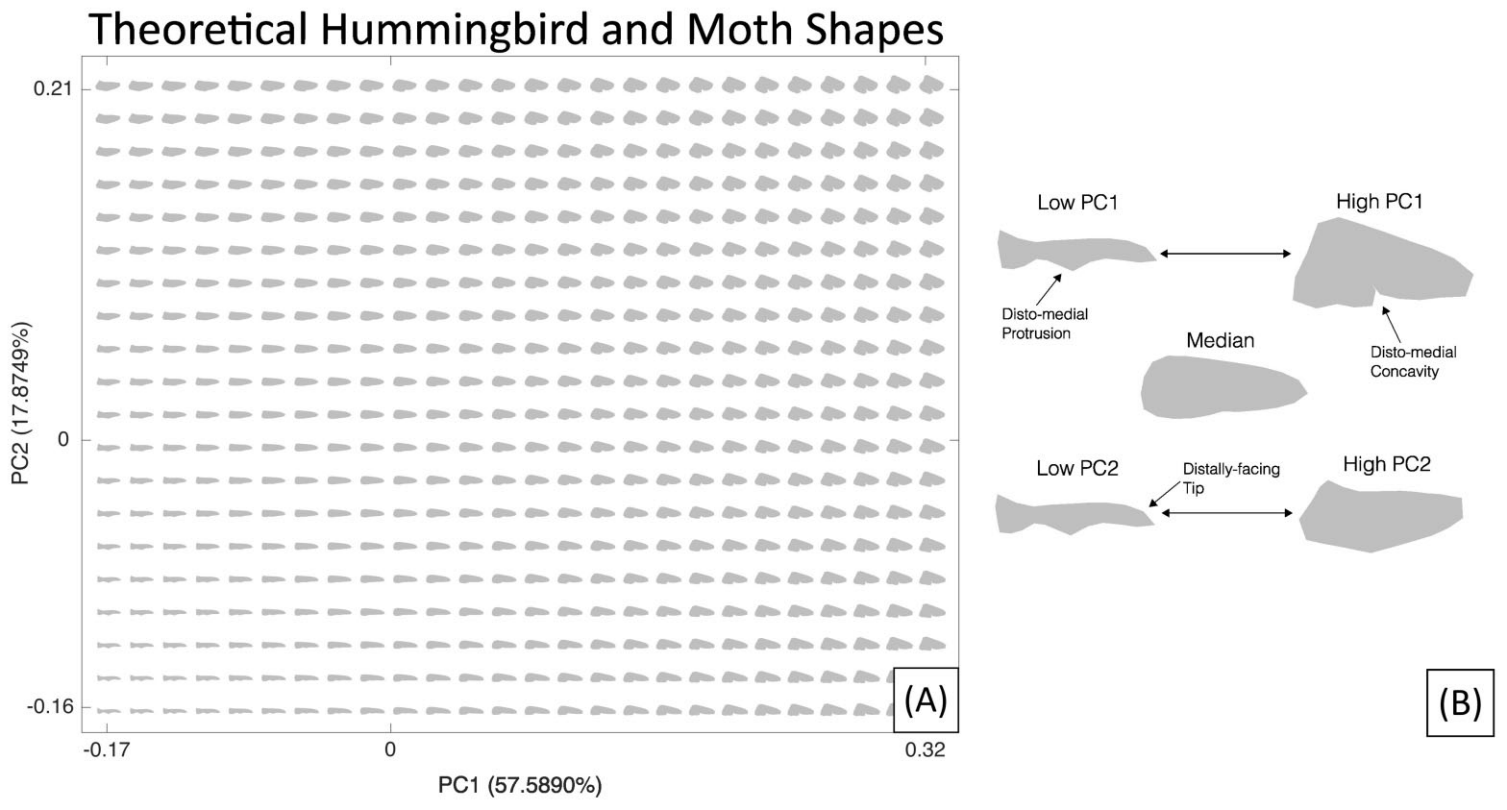
(A) theoretical shapes and optimality surface for hummingbirds, *Hemaris*, and *Macroglossum* moths. (B) annotated example of theoretical planforms illustrating the extremes of the two main axes of variation with notable morphological features highlighted.

The first two principal components of shape for the theoretical hummingbird and hawkmoth planforms represent a combined 75% of total disparity, with the first component (PC1) accounting for more than half of variance (Fig. 4A). This component describes both chord-breadth and roundedness of theoretical planform shapes. Shapes with minimum PC1 values exhibit a thinner planform with a shorter root chord, a pronounced anterior aspect and a protrusion at the distal midpoint of the planform. As theoretical shape changes along this axis, the breadth of the root chord increases, the anterior aspect is flattened, and the disto-medial protrusion is replaced with a concavity. The second component (PC2), approximately 18% of variance, describes the breadth of the chord along its length. For theoretical shapes with minimum PC2 values the chord is thin, broadening with higher values. This has the effect of flattening the trailing edge in high PC2 and low PC1 theoretical planforms; the trailing edge concavity remains in theoretical shapes with high PC1 and 2 values (Fig. 4B). PC2 also corresponds with the flexion of the planform tip, with a distally facing tip in low PC2 planforms and slight anterior flexion in the highest PC2 planforms. The median theoretical planform exhibits a broad root chord, narrowing gradually to the tip, retaining a convex profile along the entire perimeter.

### Functional performance

In performance space for hummingbird and hawkmoth wings, there is an apparent trade-off between second moment of area and pitch agility (Fig. 2A, B). For second moment of area, the region of highest performance resides at minimum values of PC1 and 2, corresponding with lowest values for pitch agility. A second region of high performance exists in the alternate corner of the plot, also corresponding with lower values in the pitch agility surface. The area of highest agility is immediately adjacent to the lowest region for moment of area, resulting in a steep transition in relative performance between the highest regions in the two metrics. This is indicative of a possible antagonistic functional trade-off.

**Fig. 5:**
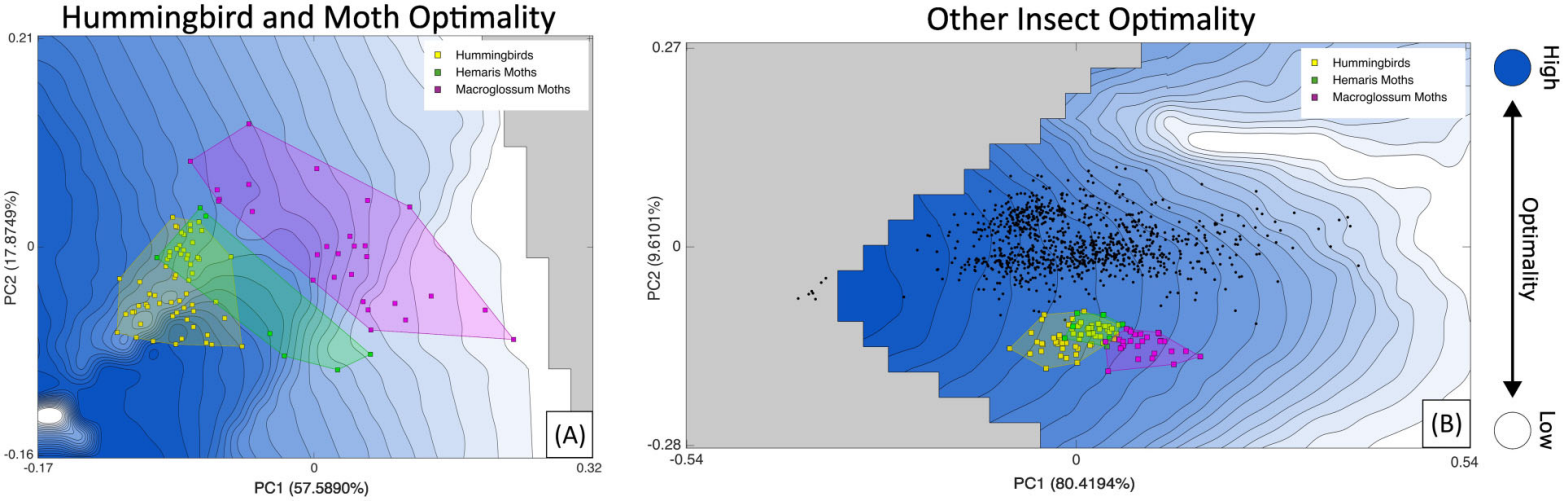
(A) Optimality surface generated from theoretical hummingbird and moth planform shapes and (B) alongside other insect groups. Empirical taxa are projected in both spaces with convex hulls. Hummingbirds in yellow, *Hemaris* moths in green and *Macroglossum* moths in purple.

The Pareto-ranked surface for this trade-off reveals a landscape characterized by three higher regions (Fig. 5A). The performance peak resides in the lower left of the plot (low PC1 and 2 values) and is subtriangular in form, dipping steeply to lower PC1 and PC2 values, with a more gradual slope to higher values of PC1. This peak is connected to two other regions of high performance by a ridge which runs diagonally upwards across the plot from low PC1/2 to high PC1/2. Both other regions of high performance reside at maximum values of PC2, constrained to the lowest and highest values of PC1 respectively. The second of these lower peaks is bounded by a region of impossible space, produced through self-intersection of the theoretical planforms. The area of least optimality resides in high PC1 and low PC2 values, bounded by impossible space, with a second pocket of low performance immediately adjacent to the peak. A valley of suboptimal space runs diagonally across the plot, following the higher PC2 edge of the ridge. The highest-performing theoretical shape has a thin root chord, lacking anterior protrusion, a distally facing tip and a slight scalloping to the trailing edge.

The Pareto-ranked trade-off surface for the other sampled insect groups (Fig. 5B) produces a single peak, situated at the lowest possible region of PC1 and median PC2 values, bounded on the left by a recess of impossible space. Optimality decreases with higher PC1 values, following the contours of impossible space. The area of lowest performance resides at the highest PC1 and median PC2 values. The highest performing theoretical shapes for this analysis are rodlike in profile, with a slight increase in chord breadth at the base and tip and concavity along the trailing edge.

### Empirical shape

By projecting empirical hummingbird and hawkmoth planforms into performance space, it is possible to determine how these groups differ in their degree of functional constraint. When projected into a space without other insect groups (Fig. 5A), hummingbirds, *Hemaris* and *Macroglossum* moths exhibit separation, with only a single *Hemaris* taxa, the Broad-bordered Bee Hawkmoth (*Hemaris fuciformis*), overlapping with the hummingbird convex hull. Hummingbirds occupy the lowest PC1 values, with the thinnest root chord, while *Macroglossum* moths occupy the largest area of morphospace with the highest PC1 values and do not overlap with either group. No empirical wing planform occupies the peak. The highest-performing forms observed are those of hummingbirds, but the majority of hummingbird taxa reside in the suboptimal valley to higher PC2 values of the peak. As the underlying performance space is Euclidian, relative distance from the peak (a performance value of 1) can be interpreted as the difference in shape similarity to the most optimal form (Walters et al. 2026). The difference between the value of the highest performing studied hummingbird planform, the White-necked Jacobin (*Florisuga mellivora*), and the peak value is 0.0893, representing an approximate convergence in shape of 91%. Both *Hemaris* and *Macroglossum* moths occupy lower regions of morphospace, with the highest performing representatives of each group situated in a region halfway up the peak.

When projected into morphospace with other insect groups (Fig. 5B), hummingbirds and hawkmoths plot in a small area situated toward the centre of the graph with median PC1 and slightly lower than median PC2 values. This region of occupation is much smaller than that of the other sampled insect groups. Group distribution mirrors that of the previous morphospace (Fig. 5A), with hummingbirds clustered around PC1 = 0 followed by *Hemaris* and then *Macroglossom* moths to slightly higher PC1 values, though there is more overlap between groups. All but three *Hemaris* moths and a single *Macroglossum* taxa, the Large Hummingbird Hawkmoth (*Macroglossum faro*), overlap with the convex hull of hummingbirds. Hummingbirds once again occupy the closest region to the peak, though this region remains unoccupied. Examples from all three sampled insect lineages occupy higher-performance regions of morphospace than the sampled hummingbirds or hawkmoths. The closest insect to the peak is the Leaf-mining Moth, *Acrocercops transecta*, followed by several neuropteran planforms which overlie on the adjacent impossible space.

## Discussion

Proximity in shape space between hummingbirds, *Hemaris* and *Macroglossum* moths, to the exclusion of most other sampled insects (Fig. 5B), demonstrates that there is convergence upon this planform shape. In birds alone, this form appears to be highly constrained by function (Walters et al. 2026), though when compared with other sampled groups, hummingbird and hawkmoth planforms exhibit lower performance, suggesting looser functional constraint. This suggests that, while they are likely a driver of shape in part, the tested metrics alone cannot explain the shape of hummingbird wings. Since a large portion of hummingbird flight is active hovering, it is likely that an overall optimal wing shape for hummingbirds would be constrained away from the best performing shape for the trade-off tested by this analysis. As a result, this analysis alone is insufficient to test for overall planform shape optimality. This is complicated however by the sampled members of Hymenoptera, which perform hovering flight and plot closer to the observed performance peak. Part of this discrepancy may be accounted for by differences in body size or other factors pertaining to organism biology.

It may be the case that function is a primary constraint on hummingbird planform shape, but that the highest performance planform shapes lie outside the realm of possibility for avians. There are a multitude of biological factors which may restrict the evolution of higher performance planforms, including those specific to birds such as the material properties of feathers. Factors intrinsic to vertebrate biology, such as the attachment of muscles from the wing to an internal bony scaffold, may also influence shape, leading to a broader basal wing chord. The endothermic nature of birds likely also imposes strong constraints on hummingbirds. Hovering flight is metabolically intensive, requiring elevated nutrient intake (Weis-Fogh 1972; Suarez et al. 1986; Suarez 1992), a factor compounded by the need to maintain a constant elevated internal body temperature.

The lack of clear separation of hummingbird wing planforms across the main principal components of shape suggests that the sexual dimorphism recorded in some hummingbird groups has not been sampled by the dataset (Fig. 3B); however, the effect of dimorphism on shape analyses should not be understated. This disparity is likely due to the use of single wings as representatives for each species, which may not fully capture the variation seen at a species level. As a result, it is not possible fully separate interspecific and dimorphism-related variation within the dataset for any members of tribes with known dimorphism. Though all wings used in this analysis were fully spread and complete, this is potentially compounded by the way that wings were spread and prepared when collected. A sample set which includes both male and female examples of the same taxa could provide a better overview of the degree to which use of the wing for display alters morphology. Lack of observed phylogenetic signal may indicate that the genetic relationship to shape analyses is minimal, though the true phylogenetic signal of dimorphism is likely underrepresented by the sampling method employed. Sampling single individuals from both sexes from within sexually dimorphic tribes will obscure the relationship between phylogeny and shape, which is likely higher than reported here. The even distribution of taxa regardless of sex suggests that dimorphism is not a primary driver of shape disparity within this dataset, though its effects cannot be discounted.

The lack of strong functional constraint in hummingbirds may relate to the limits of the vertebrate bauplan, but this does not explain why both groups of hawkmoths examined converge upon the hummingbird shape instead of developing wings more like those of other insects. Feeding constraints may explain this result. Efficient hovering is essential for effective nectivorous feeding (Warrick et al. 2012a; Wester 2013), on which these groups depend, and may influence wing shape. This also pertains to hummingbirds, alongside other co-options of the wing for chasing rivals and display purposes. Several taxa across insects, including other members of Lepidoptera, possess wings with greater observed constraint than the *Hemaris* and *Macroglossum* moths, suggesting that the insect Bauplan can produce shapes with greater functional performance for the tested metrics. The lack of truly optimal insect planforms in this analysis is likely the result of sampling coverage, due to a lack of available data for groups such as Odonata and Diptera.

Convergence upon a hummingbird-like planform within the sampled hawkmoths appears to represent evolution away from an adaptive functional optimum, the opposite of the pattern expected for animals that depend on rigorous activities such as flight. In new world hawkmoths (members of the *Hemaris* genus), a potential explanation for this shift may be niche competition. As both animal groups exhibit the same strategy and exist in the same environment, the large energy budget required may drive competition between these groups. Past a certain threshold, a hummingbird-like wing may be more advantageous than one with greater functional performance. This is further evidenced by co-option of the better-performing lepidopteran 4-winged flight apparatus for a 2-winged flight mode more reminiscent of hummingbirds (Warfvinge et al. 2021; Lionetti et al. 2022; Xue et al. 2024).

Competition with hummingbirds, however, does not explain the shape of *Macroglossum* planforms, as these taxa are restricted to the old world and have no extant overlap with hummingbirds. The last occurrence of hummingbirds in the old world is from the Oligocene of Europe approximately 30ma (Bochenski and Bochenski 2008). *Macroglossum* planforms do exhibit less similarity to hummingbirds than those of *Hemaris* moths, occupying a larger region of shape space which overlaps minimally with hummingbirds in both analyses. *Macroglossum* moths are therefore less constrained by the factors that drive similarity between the other two groups, though what does drive the shape of their wings is unclear.

## Conclusion

Theoretical morphospace permits direct comparison between non-homologous structures demonstrating that shape convergence between hummingbird and hawkmoth planforms is not primarily driven by functional constraint. Hummingbird planforms exhibit middling functional performance when compared with insects, in contrast to the high performance suggested by analysis of birds alone. This highlights the value in discussing function in broad, taxonomically diverse datasets, rather than focusing on groups in isolation. It is likely that biological constraints restrict the evolution of higher performance wing shapes in hummingbirds. In *Macroglossum* moths, function appears to have little influence on planform shape. Convergence of *Hemaris* and *Macroglossum* moths on a hummingbird-like wing planform appears to suggest adaptation away from regions of higher functional performance, contrary to the result expected from adaptationist thinking. This can potentially be explained in part due to niche constraints or competition and appears to affect moth genera differently.

## Author contributions

Conceptualization: B.W., E.J.R., P.C.J.D. Data curation: B.W., Y.L., I.F., H.G.F. Methodology: B.W., E.J.R., P.C.J.D. Software: B.W., Y.L., E.J.R., P.C.J.D. Formal analysis: B.W. Visualization: B.W. Supervision: E.J.R., P.C.J.D. Writing—original draft: B.W. Writing—review and editing: B.W, E.J.R., P.C.J.D.

## Data Availability

All data generated by this study are deposited on Figshare and are accessible without restriction at https://figshare.com/s/b808b25356b02ea7fa12. The Lepidoptera dataset used in analysis is available at 10.6084/m9.figshare.31828372, with both the Neuroptera and Hymenoptera datasets acquired from their respective papers (Liu et al. 2024; Fadel et al. 2025). Supplementary Information is available online at https://figshare.com/s/b808b25356b02ea7fa12. The base code used for analysis and Fig. generation is available on Github, accessible at Deakin, W., Rayfield, E., & Donoghue, P. theofun (Version 0.0.1) [Computer software]: https://github.com/Bristol-Palaeobiology/theofun). All executable files for the analysis are provided in the supplementary material on Figshare under Supplementary Code 1, accessible at https://figshare.com/s/b808b25356b02ea7fa12. All files are freely available for download without restriction.

## Notes

From the symposium "Dinosaurs to Drones: Comparative Approaches to Understanding Flight" presented at the annual meeting of the Society for Integrative and Comparative Biology, January 3-7, 2026.

## References

[bib1] Adams D, Otárola-Castillo E. 2013. Geomorph: an R package for the collection and analysis of geometric morphometric shape data. Methods Ecol Evol. 4:393–9.

[bib2] Amorim F . 2020. Are the new world hummingbird-hawkmoths functional equivalents of hummingbirds?’. Ecology. 101:1–4. 10.1002/ecy.3161 33448357

[bib3] Berks H, Milla Carmona P, Donoghue P, Rayfield E. 2025. The evolution of herbivory, not terrestrialization, drove morphological change in the mandibles of palaeozoic tetrapods. Evol J Linnean Soc. 4:kzaf004.

[bib4] Bochenski Z, Bochenski Z. 2008. An old world hummingbird from the Oligocene: a new fossil from polish Carpathians. J Ornithol. 149:211–6.

[bib5] Chai P, Altshuler D, Stephens D, Dillon M. 1999. Maximal horizontal flight performance of hummingbirds: effects of body mass and molt. Physiol Biochem Zool. 72:145–55. 10068617 10.1086/316652

[bib6] Clark C, Dudley R. 2009. Flight costs of long, sexually selected tails in hummingbirds. Proc R Soc Lond Ser B. 276:2109–15. 10.1098/rspb.2009.0090 PMC2677254 19324747

[bib7] Deakin W, Anderson P, den Boer W, Smith T, Hill J, Rücklin M, Donoghue P, Rayfield E. 2022. Increasing morphological disparity and decreasing optimality for jaw speed and strength during the radiation of jawed vertebrates. Sci Adv. 8:eabl3644. 35302857 10.1126/sciadv.abl3644 PMC8932669

[bib8] Dickson B, Clack J, Smithson T, Pierce S. 2021. Functional adaptive landscapes predict capacity at the origin of limbs. Nature. 589:242–5. 33239789 10.1038/s41586-020-2974-5

[bib9] Dickson B, Pierce S. 2019. Functional performance of turtle humerus shape across an ecological adaptive landscape. Evolution. 73:1265–77. 31008517 10.1111/evo.13747

[bib10] Ellington C . 1984. The aerodynamics of hovering insect flight. II. Morphological parameters. Philos Trans R Soc B Biol Sci. 305:17–40.

[bib11] Fadel I, Milla Carmona P, Liu Y, Rayfield E, Donoghue P. 2025. Morphological constraints in hymenopteran forewings limit flight efficiency optimization. R Soc Open Sci. 12:250224. 40727405 10.1098/rsos.250224 PMC12303117

[bib12] Goldberg D . 1989. Genetic algorithms in search, optimization, and machine Learning. Addison-Wesley Longman Publishing Co. Inc. Reading, MA.

[bib13] Gould S, Lewontin R. 1979. The spandrels of San Marco and the Panglossian paradigm: A critique of the adaptationist programme. Proc R Soc Lond Ser B. 205:581–98. 42062 10.1098/rspb.1979.0086

[bib14] Griffin B, Keating J, Milla Carmona P, Johanson Z, Dearden R, Donoghue P, Rayfield E. 2025. Evolution of chondrichthyan jaw morphology, from ecological generalists to specialists. Paleobiology:1–14.

[bib15] Hackett S, Kimball R, Reddy S, Bowie R, Braun E, Braun M, Chojnowski J, Cox A, Han K, Harshman J et al. 2008. A phylogenomic study of birds reveals their evolutionary history. Science. 320:1763–8. 18583609 10.1126/science.1157704

[bib16] Haider N, Shahzad A, Qadri M, Shams T. 2021. Aerodynamic analysis of hummingbird-like hovering flight. Bioinspir Biomim. 16:066018. 10.1088/1748-3190/ac28eb 34547732

[bib17] Halloway A, Whelan C, Brown J. 2018. The hummingbird and the hawk-moth: species distribution, geographical partitioning, and macrocompetition across the United States. Biorxiv.212894.

[bib18] Hally D . 1987. Calculation of the moments of polygons. Defence Research Establishment Atlantic. Suffield Ralston (Alberta).

[bib19] Harvey C, Baliga V, Wong J, Altshuler D, Inman D. 2022. Birds can transition between stable and unstable states via wing morphing. Nature. 603:648–53. 35264798 10.1038/s41586-022-04477-8 PMC8942853

[bib20] Ingersol R, Lentink D. 2018. How the hummingbird wingbeat is tuned for efficient hovering. J Exp Biol. 221:178228. 10.1242/jeb.178228 30323114

[bib21] Lionetti S, Hedrick T, Li C. 2022. Aerodynamic explanation of flight speed limits in hawkmoth-like flapping-wing insects. Phys Rev Fluids. 7:093104.

[bib22] Liu Y, Deakin W, Rayfield E, Donoghue P. 2024. Theoretical morphospace analysis of neuropteran wings reveals little evidence of optimization for flight performance. Evol J Linnean Soc. 3:kzae019.

[bib23] Lockwood R, Swaddle J, Rayner J. 1998. Avian wingtip shape reconsidered: Wingtip shape indices and morphological adaptations to migration. J Avian Biol. 29:273–92.

[bib24] McGhee G . 2006. The geometry of evolution: adaptive landscapes and theoretical morphospace. Cambridge: Cambridge University Press.

[bib25] Norberg U . 1987. Wing form and flight mode in bats. In Fenton M., Racey P., Rayner J. Recent advances in the study of bats:Cambridge: Cambridge University Press. p. 43–56.

[bib26] Norberg U . 1990. Vertebrate flight: mechanics, physiology, morphology, ecology and evolution. Berlin, Heidelberg: Springer Science and Business Media.

[bib27] Norberg U . 2002. Structure, form and function of flight in engineering and the living world. J Morphol. 252:52–81. 11921036 10.1002/jmor.10013

[bib28] Norberg U, Rayner J. 1987. Ecological morphology and flight in bats (Mammalia; Chiroptera): wing adaptations, flight performance, foraging strategy and echolocation. Phil Trans R Soc B. 316:335–427.

[bib29] Pearson O . 1950. The metabolism of hummingbirds. The Condor. 52:145–52.

[bib30] Pennycuick C . 1975. Mechanics of flight. Avian Biology. 5:1–75.

[bib31] Pennycuick C . 1989. Bird flight performance: A practical calculation manual. United Kingdom: Oxford University Press.

[bib32] Rawson J, Deakin W, Stubbs T, Smith T, Rayfield E, Donoghue P. 2024. Widespread convergence towards functional optimization in the lower jaws of crocodile-line archosaurs. Proc R Soc B. 291:20240720. 10.1098/rspb.2024.0720 PMC11335402 39163982

[bib33] Rayner J . 1988. Form and function in avian flight. Current Ornithol. 5:1–66.

[bib34] Revell L . 2011. Phytools: an R package for phylogenetic comparative biology (and other things). Methods Ecol Evol. 3:217–23.

[bib35] Saville D . 1957. Adaptive evolution in the avian wing. Evolution. 11:212–224.

[bib36] Schindelin J, Arganda-Carreras I, Frise E, Kaynig V, Longair M, Pietzsch T, Preibisch S, Rueden C, Saalfeld S, Schmid B et al. 2012. Fiji: An open-source platform for biological-image analysis. Nat Methods. 9:676–82. 22743772 10.1038/nmeth.2019 PMC3855844

[bib37] Segre P, Dakin R, Read T, Straw A, Altshuler D. 2016. Mechanical constraints on flight at high elevation decrease maneuvering performance of hummingbirds. Curr Biol. 26:3368–74. 27939316 10.1016/j.cub.2016.10.028

[bib38] Stayton T . 2019. Performance in three shell functions predicts the phenotypic distribution of hard-shelled turtles. Evolution. 73:720–34. 30820948 10.1111/evo.13709

[bib39] Stiles G, Altschuler D, Dudley R. 2005. Wing morphology and flight behaviour of some North American hummingbird species. The Auk. 122:872–86.

[bib40] Suarez R . 1992. Hummingbird flight: sustaining the highest mass-specific metabolic rates among vertebrates. Experientia. 48:565–70. 1612136 10.1007/BF01920240

[bib41] Suarez R, Brown G, Hochachka P. 1986. Metabolic sources of energy for hummingbird flight. Am J Physiol-Regul Integr Comp Physiol. 251:R537–42. 10.1152/ajpregu.1986.251.3.R537 3752286

[bib42] Swaddle J, Lockwood R. 2003. Wingtip shape and flight performance in the european starling *Sturnus vulgaris*. Ibis. 145:457–64.

[bib43] Taylor G, Thomas A. 2014. Evolutionary Biomechanics: Selection, Phylogeny, and Constraint. Oxford University Press: United Kingdom.

[bib44] The MathWorks, Inc. 2022. MATLAB Version: 9.13.0 (R2022b). Massachusetts: The MathWorks, Natic

[bib45] Tobalske B . 2010. Hovering and intermittent flight in birds. Bioinspir Biomim. 5:045004. 21098953 10.1088/1748-3182/5/4/045004

[bib46] Tobalske B, Warrick D, Clark C, Powers D, Hedrick T. Hyder G, Biewener A. 2007. Three-dimensional kinematics of hummingbird flight. J Exp Biol. 210:2368–82. 17575042 10.1242/jeb.005686

[bib47] Walters B, Liu Y, Rayfield E, Donoghue P. 2026. Theoretical morphology of avian wing planform reveals variable optimisation to flight style. Nat Commun. 17:3902. 41917011 10.1038/s41467-026-70692-w PMC13128903

[bib48] Warfvinge K, Johansson C, Hedenström A. 2021. Hovering flight in hummingbird hawkmoths: kinematics, wake dynamics and aerodynamic power. J Exp Biol. 224:230920. 10.1242/jeb.230920 34042974

[bib49] Warham J . 1977. Wing loadings, wing shapes, and flight capabilities of procellariiformes. NZ J Zool. 4:73–83.

[bib50] Warrick D, Hedrick T, Fernández M, Tobalske B, Biewener A. 2012b. Hummingbird flight. Curr Biol. 22:R472–7. 22720675 10.1016/j.cub.2012.04.057

[bib51] Warrick D, Tobalske B, Powers D. 2005. Aerodynamics of the hovering hummingbird’. Nature. 435:1094–7. 15973407 10.1038/nature03647

[bib52] Warrick D, Tobalske B, Powers D. 2009. Lift production in the hovering hummingbird. Proc R Soc B. 276:3747–52. 10.1098/rspb.2009.1003 PMC2817280 19656789

[bib53] Warrick D, Tobalske B, Powers D, Dickinson M. 2012a. The aerodynamics of hummingbird flight. 45^th^ AIAA Aerospace Sciences Meeting and Exhibit 10.2514/6.2007-41.

[bib54] Wester P . 2013. Feeding on the wing: hovering in nectar drinking Old World birds—more common than expected. Emu Austral Ornithol. 114:171–83.

[bib55] Weis-Fogh T . 1972. Energetics of hovering flight in hummingbirds and in Drosophila. Journal of Experimental Biology. 56:79–104.

[bib56] Wilcox S, Clark C. 2022. Sexual selection for flight performance in hummingbirds. Behav Ecol. 33:1093–106.

[bib57] Xue Y, Cai X, Liu H. 2024. Aerodynamics and stability of hawkmoth forward flight with flexible wing hinge. Phys Rev Fluids. 9:063101.

